# Efficacy of injectable platelet-rich fibrin in the erosive oral lichen planus: a split-mouth, randomized, controlled clinical trial^*^

**DOI:** 10.1590/1678-7757-2021-0180

**Published:** 2021-10-04

**Authors:** Ebru SAGLAM, Zeliha Betul OZSAGIR, Tugba UNVER, Suzan Bayer ALINCA, Ali TOPRAK, Mustafa TUNALI

**Affiliations:** 1 Health Sciences University Faculty of Dentistry Department of Periodontology Istanbul Turkey Health Sciences University, Faculty of Dentistry, Department of Periodontology Istanbul, Turkey.; 2 Bezmialem Vakif University Faculty of Dentistry Department of Maxillofacial Radiology Istanbul Turkey Bezmialem Vakif University, Faculty of Dentistry, Department of Maxillofacial Radiology, Istanbul, Turkey.; 3 Kecioren Osmanli Public Oral Health Center Oral and Maxillofacial Surgery Ankara Turkey Kecioren Osmanli Public Oral Health Center, Oral and Maxillofacial Surgery, Ankara, Turkey.; 4 Bezmialem Vakif University Faculty of Medicine Department of Biostatistics and Medical Informatics Istanbul Turkey Bezmialem Vakif University, Faculty of Medicine, Department of Biostatistics and Medical Informatics, Istanbul, Turkey.; 5 Canakkale Onsekiz Mart University Faculty of Dentistry Department of Periodontology Canakkale Turkey Canakkale Onsekiz Mart University, Faculty of Dentistry, Department of Periodontology, Canakkale, Turkey.

**Keywords:** Corticosteroid, Oral lichen planus, Platelet-rich fibrin, Patient reported outcome measures, Wound healing

## Abstract

**Objective:**

Our study compared the effects of injectable platelet-rich fibrin (i-PRF) with those of corticosteroids in the treatment of erosive oral lichen planus (EOLP).

**Methodology:**

This split-mouth study included 24 individuals diagnosed histopathologically with bilateral EOLP. One bilateral lesion was injected with i-PRF, whereas the other was injected with methylprednisolone acetate in four sessions at 15-day intervals. Visual analog scale (VAS) for pain and satisfaction, oral health impact profile scale-14, and the lesion size were used.

**Results:**

The intragroup comparisons showed a significant decrease in VAS-pain and lesion size in both the i-PRF group (from 81.88±17.74 to 13.33±18.34, and from 4.79±0.41 to 1.88±1.08, respectively) and the corticosteroid group (from 80.21±17.35 to 23.33±26.81, and from 4.71±0.46 to 2.21±1.35, respectively) in the 6th month compared to baseline (p<0.001). Moreover, VAS-satisfaction increased significantly in both the i-PRF group (from 26.67±17.8 to 85.63±16.24) and the corticosteroid group (from 28.33±17.05 to 74.38±24.11) in the 6th month compared to baseline (p<0.001). However, no significant difference in any value occurred in the intergroup comparisons.

**Conclusion:**

In patients with EOLP, both methods decreased pain and lesion size similarly, and both increased satisfaction. Therefore, the use of i-PRF may be considered an option in cases refractory to topical corticosteroid therapy. Biochemical and histopathological studies are required to reveal the mechanism of i-PRF action in EOLP treatment.

## Introduction

Oral lichen planus (OLP) is a chronic inflammatory immune mediated disease of unknown cause, in which T lymphocytes attack multi-layer flat epithelial cells.^1,2^ Most infiltrating lymphocytes seen in OLP are CD8+, so the condition probably results from a cytotoxic autoimmune response.^3^ Furthermore, CD4+ T lymphocytes increase the cytotoxicity of CD8+ lymphocytes by infiltrating OLP lesions. The buccal mucosa, tongue and gingiva are the most frequently affected regions in the oral cavity, and the lesions can occur symmetrically, bilaterally, or unilaterally. Reticular and papular OLP lesions are often asymptomatic; however, atrophic and erosive oral lichen planus (EOLP) forms can negatively affect patients’ quality of life, causing sensitivity, burning symptoms, and discomfort.^4^

The treatment for OLP lesions include different pharmacological agents, such as corticosteroids, immunosuppressives, retinoids and metronidazole.^5^ Corticosteroid treatment, in particular, can lead to several side effects, such as pain, bleeding, ulceration, secondary infections, perilesional linear atrophy, hypopigmentation, allergic reactions, calcification and granuloma.^4,5^ Consequently, medical treatment may include different alternative therapies, such as biostimulation with diode laser, photodynamic therapy based on methylene blue, psoralen and ultraviolet A therapy (a form of photochemotherapy treatment), ozone therapy and herbal remedies with anti-inflammatory properties (e.g., aloe vera, lycopene).^1^

Platelet-rich fibrin (PRF) is a three-dimensional fibrin network that accelerates wound healing, immunity and neovascularization. It contains host immune defense cells (leukocytes) and promotes three important stages of wound healing: angiogenesis, immune response and epithelial proliferation.^6^ In one study comparing PRF and connective tissue graft to treat gingival recession, the levels of gingival crevicular fluid proinflammatory markers (interleukin (IL)-1β and matrix metalloproteinase-8) were significantly lower in the PRF group.^7^ Similar to conventional PRF, injectable PRF (i-PRF) increases the number of leukocytes and stimulates growth factor release.^8^ In another study, direct injection of the lesion with growth factor-rich plasma, which is a first generation blood product and contains anticoagulants, decreased pain and improved quality of life in patients with OLP.^9^

The i-PRF is an autogenous product that acts as a growth factor release system involving transforming growth factor- β (TGF-β), platelet-derived growth factor (PDGF), and vascular endothelial growth factor (VEGF).^10,11^ It has been shown to have an important regenerative role in human skin fibroblasts.^12^ The i-PRF exhibits a supportive regenerative property for osteoblastic differentiation and reparative dentin stimulation in human dental pulp cells,^13^ besides attenuating the inflammatory state induced by lipopolysaccharides.^13^ Due to its bioactive nature, i-PRF can be used in combination with collagen-based biomaterials to increase healing activity.^10^ Also, it can be mixed with bone grafts and used to graft bone defects in Dentistry,^11^ and to treat facial cutaneous tracts secondary to medication-related osteonecrosis of the jaw^14^ and cell mucositis of the oral cavity.^15^

Due to the numerous positive features of i-PRF, we aimed to evaluate its effect on EOLP lesions and quality of life in this present study.

## Methodology

**Figure 1:**
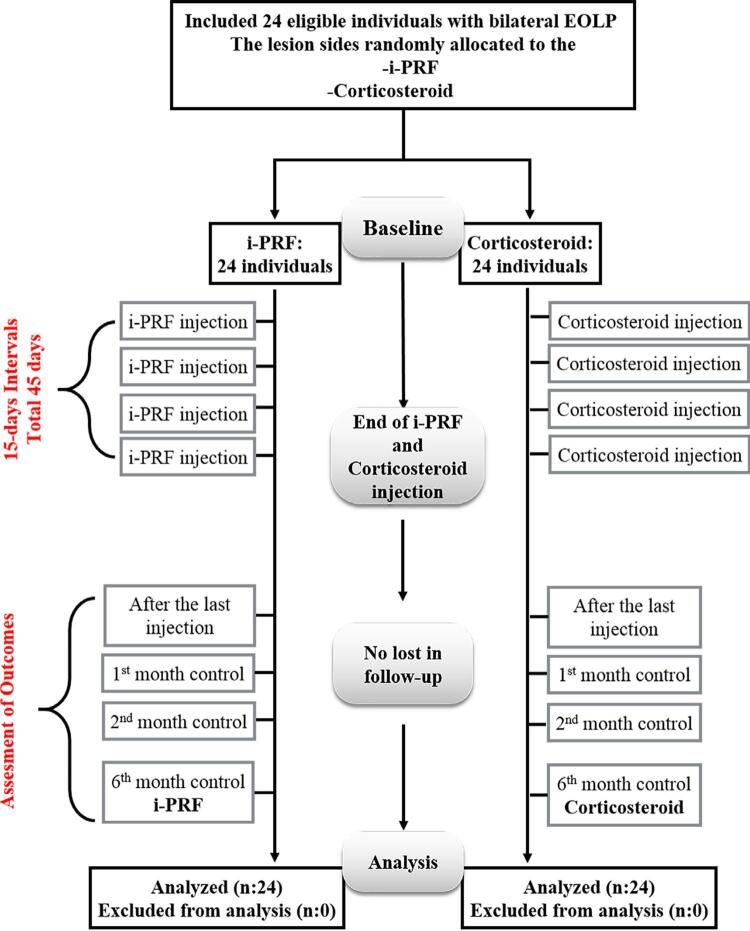
Flow chart

This randomized, controlled, prospective, split-mouth study involved patients diagnosed both clinically and histopathologically with bilateral EOLP. The study protocol was approved by the Clinical Research Ethics Committee of Bezmialem Vakıf University (2017-12/20). Individuals referred to the Periodontology Department of the Faculty of Dentistry at Bezmialem Vakıf University were informed about the treatment protocol, and written consent was obtained from the individuals in accordance with the Declaration of Helsinki. The study was registered on ClinicalTrials.gov (NCT03265093) and was conducted from June 2017 to September 2019. Figure 1 shows the study flow chart.

### Patient selection

To ensure 80% power (1 - β) with a 95% confidence interval (α=0.05), and an effect size of 1.01, and considering the lesion size scores at the 2^nd^ month (according to Thongprasom), the study size was estimated as 17 patients per group.^16^ Therefore, 24 individuals were included in case of any dropout.

Our study included systemically healthy volunteers who were diagnosed clinically and histopathologically with bilateral EOLP according to Andreasen classification^17^ and World Health Organization criteria^18^, and who were refractory to topical corticosteroid therapy. The punch biopsy method was used for histopathological evaluation, and no volunteers had received lichen planus treatment in the previous 3 months. The baseline and 2^nd^ month follow-up data for 13 patients were included in our previous pilot study.^19^

The exclusion criteria were: age younger than 18 years, pregnancy or breastfeeding, systemic disease (e.g., diabetes, Cushing’s syndrome), coagulation disorders, smoking, infectious disease (e.g., hepatitis), use of anticoagulant drugs, psychiatric problems, dysplasia upon histopathology^16^ and OLP manifestations in other mucous membranes simultaneously.

### Preparation of i-PRF

Venous blood was collected from each patient using a 20-mL syringe and placed in two plastic i-PRF tubes (10 mL each, without anticoagulant). The tubes were centrifuged with Intra-spin system, Intra-Lock centrifuge (Process for PRF, Boca-Raton, FL, EUA) at 700 rpm for 3 min (47 g force) to obtain i-PRF. The i-PRF was then drawn into dental injectors with 27 gauge needle tips in preparation for injection.

### Application protocol

A topical anesthetic gel containing 20% benzocaine (VISION Pat Gel; Anadolu Dis Deposu, Istanbul, Turkey) was applied to the EOLP region before the procedure was started. In the same session, one of the bilateral EOLP lesions was injected with i-PRF and the other with methylprednisolone acetate. Both injections were administered during four sessions and had a 15-day interval between each session.

*i-PRF Group*; i-PRF obtained as a result of centrifugation was drawn into a dental injector with 27-gauge needle tip in preparation for injection. It was injected at four different endpoints at the periphery of the lesion, as described in the study by Pinas, et al.^9^ (2017).

*Corticosteroid Group;* Methylprednisolone acetate (40 mg/mL Depo-medrol; Eczacibasi, Istanbul, Turkey) was applied with an insulin syringe (29-gauge needle) at four different endpoints into the subepithelial tissue underlying the lesion and adjacent to the normal mucosa.^20^ Each injection was 0.2 mL per session.

### Evaluation of visual analog scale-pain, visual analog scale-satisfaction, oral health impact profile-14 and thongprasom classification

The effects of pharmaceutical and alternative therapies were evaluated using the visual analog scale (VAS) for pain^21^ and satisfaction,^22^ the 14-item oral health impact profile (OHIP-14),^23^ and objective evaluation of lesion size.^24^

The participants were asked to determine the degree of complaint and their satisfaction using a 100-unit chart for VAS. In the VAS-pain scale, 0 points indicated no complaints, and 100 reflected the most severe complaint. Likewise, 0 points on the VAS-satisfaction scale indicated no satisfaction and 100 points denoted very good satisfaction. The scales were performed just before the first injection, immediately after the last injection session, and at the 1^st^, 2^nd^, and 6^th^ months of the control sessions.

The OHIP-14 questionnaire was used to evaluate quality of life consisting in seven domains and with two questions in each domain.^23^ Patients answered questions about discomfort and inadequacies regarding oral health, assigning a score between 0 and 4 for each question, therefore, a total score between 0 and 56 was estimated. The lowest score of 0 indicated a very good quality of life, whereas the highest score of 56 reflected a very poor quality of life. Scores higher than 14 indicated poor oral health-related quality of life.^23^ The scale was adapted for Turkish patients by Mumcu, et al.^25^ (2006), who reported a Cronbach’s α-value of 0.94. The questionnaire was administered immediately before the first injection, and at the 1^st^, 2^nd^, and 6^th^ month control sessions.

The width and length of the erosive lichen planus surface on both sides were marked on the abaisse tongue. The points marked were measured using a digital calliper (150 mm digital calliper, Alpha Tools^®^, Oakland, NJ, USA) with a sensitivity of 0.01 mm. The calculated surface areas were evaluated according to the method by Thongprasom, et al.^24^ (2003). Specifically, a score between 0 and 5 points was assigned (score 0: no lesion, score 1: only white stria, score 2: < 1 cm^2^ white line with erythematous area, score 3: > 1 cm^2^ white line with erythematous area, score 4: < 1 cm^2^ white line with erosive area, score 5: > 1 cm^2^ white line with erosive area). The evaluation was performed just before the first injection, and at the 1^st^, 2^nd^, and 6^th^month control sessions.

### Randomization

The lesions of the patients were randomly divided into two groups by an independent researcher (T.U.) using a computer-assisted randomization table (www.randomizer.org; Copyright^©^ 1997–2011 by Geoffrey C. Urbaniak and Scott Plous). Assignments were hidden from the physician performing the treatment (Z.B.Ö.) until the first treatment session, from the physician recording the measurements throughout the study, and from patients throughout the study.

### Statistical analysis

Descriptive statistics were used to define continuous variables, whereas the Shapiro-Wilk test was used to evaluate the normality of data distribution. The Mann-Whitney U test was conducted to assess intergroup comparisons, and, the Friedman test was used to the intragroup comparisons values (time-varying multiple dependent variables), followed by *post-hoc* pairwise comparisons with the Bonferroni-corrected Wilcoxon’s signed-rank test. Parameters with p<0.05 were considered significant. In the case of Bonferroni-corrected Wilcoxon tests of the VAS-pain and VAS-satisfaction scores, p<0.005 was considered significant, also, in the case of Thongprasom and OHIP-14 assessments, p<0.008 was considered significant. MedCalc Statistical Software version 12.7.7 (MedCalc Software bvba, Ostend, Belgium; http://www.medcalc.org) was used for the analyses.

## Results

**Figure 2:**
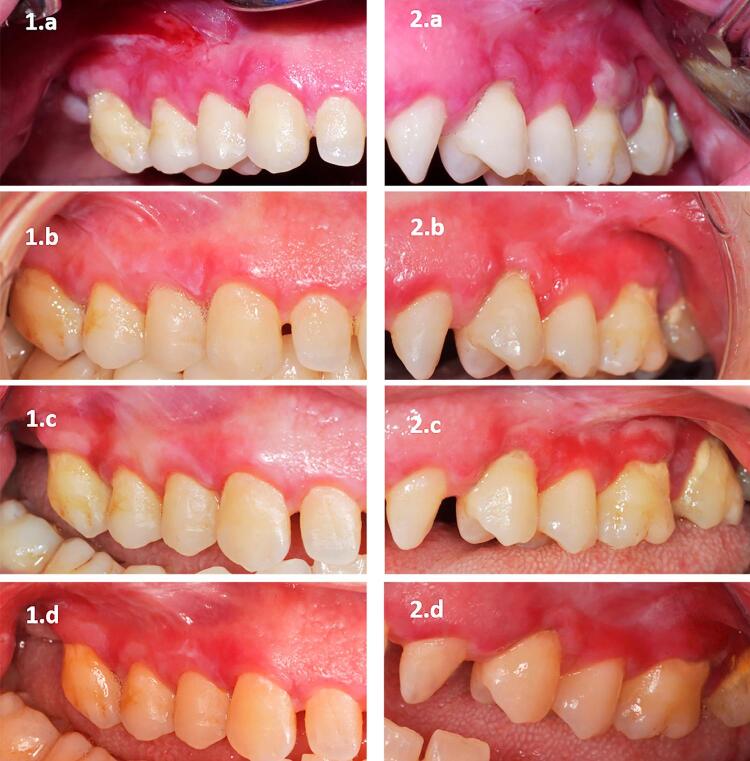
The picture comparing (1) injectable platelet-rich fibrin (i-PRF) and (2) corticosteroid injection (a) at baseline, and at the (b) 1st, (c) 2nd, and (d) 6th months after treatment

Our study included 24 patients (14 women and 10 men) between 34 and 76 years old (mean: 52.25 years) with bilateral EOLP. To the best our knowledge, the subjects reported no systemic side effects during the injections or the follow-up period. Figure 2 shows the intraoral view of a patient with OLP before and after the injections.

The intergroup comparison showed no significant difference between the i-PRF group and the corticosteroid group regarding VAS-pain values (p>0.05 by the Mann-Whitney U test). On the other hand, the intragroup comparisons showed a statistically significant difference among all time measurements of the VAS-pain values in both i-PRF and corticosteroid groups (p<0.05 by the Friedman test). According to the *post-hoc* pairwise comparisons, the median values after the last injection and at 1^st^, 2^nd^and 6^th^months were statistically lower than the baseline values (p<0.005 by the Wilcoxon test with Bonferroni correction) when comparing VAS-pain levels of the i-PRF and corticosteroid groups within each group. The 1^st^and 2^nd^ month VAS-pain levels were significantly lower than the levels after the last injection in both groups, and the 6^th^ month VAS-pain levels in the i-PRF group were significantly lower than those after the last injection. Moreover, the 2^nd^ month VAS-pain levels in the i-PRF group were significantly lower than the 1^st^month levels (p<0.005 by the Wilcoxon test with Bonferroni correction; Table 1).

**Table 1:** Comparisons of VAS-pain and VAS-satisfaction values with intragroup and intergroup

	VAS-pain			VAS-satisfaction Levels		
	i-PRF Group	Corticosteroid Group		i-PRF Group	Corticosteroid Group	
	Mean±SD	Mean±SD	p1	Mean±SD	Mean±SD	p1
	Med (Min-Max)	Med (Min-Max)		Med (Min-Max)	Med (Min-Max)	
Baseline	81.88±17.74	80.21±17.35	0.666	26.67±17.8	28.33±17.05	0.691
	85 (40-100)	80 (40-100)		27.5 (0-70)	30 (0-70)	
After the Last Injection	37.92±25.66^a^	41.25±23.97^a^	0.625	62.92±23.63^a^	61.04±22.02^a^	0.728
	40 (0-100)	40 (0-100)		60 (10-100)	60 (20-100)	
1^st^ month	19.79±18.15^a,b^	20.83±17.61^a,b^	0.784	79.17±18.57^a,b^	76.67±17.8^a,b^	0.565
	20 (0-50)	20 (0-50)		85 (40-100)	80 (40-100)	
2^nd^ month	8.75±10.96^a,b,c^	12.29±14.37^a,b^	0.436	87.71±11.61^a,b^	84.38±12.96^a,b^	0.382
	2.5 (0-40)	10 (0-50)		90 (60-100)	80 (50-100)	
6^th^ month	13.33±18.34^a,b^	23.33±26.81^a^	0.222	85.63±16.24^a,b^	74.38±24.11^a^	0.072
	10 (0-60)	10 (0-90)		90 (50-100)	80 (10-100)	
p*	<0.001	<0.001		<0.001	<0.001	

*Friedman test p-value (intragroup); statistical significance (p<0.05) marked in bold.

1Mann-Whitney U test p-value (intergroup).

^a^ Statistical significance compare to baseline,

^b^ Statistical significance compare to after the last injection,

^c^ Statistical significance compare to 1st month, using the Wilcoxon test with Bonferroni-correction (p<0.005).

Abbreviations: VAS, visual analog scale; i-PRF, injectable platelet-rich fibrin; SD, standard deviation; Med, median; Min, minimum; Max, maximum.

The intergroup comparison also showed no significant difference between the i-PRF group and the corticosteroid group regarding VAS-satisfaction values (p>0.05 by the Mann-Whitney U test). The intragroup comparisons, in turn, showed a statistically significant difference among all time measurements of the VAS-satisfaction values in both the i-PRF and the corticosteroid groups (p<0.05 by the Friedman test). According to the *post-hoc* pairwise comparisons, the median values after the last injection and at 1^st^, 2^nd^ and 6^th^ months were significantly higher than the baseline median values (p<0.005 by the Wilcoxon test with Bonferroni correction) when comparing the VAS-satisfaction levels of the i-PRF and corticosteroid groups within the groups. The 1^st^ and 2^nd^ month VAS-satisfaction levels were significantly higher in both groups compared to levels after the last injection, whereas the 6^th^ month VAS-satisfaction levels in the i-PRF group were significantly higher than those levels after the last injection (p<0.005 by the Wilcoxon test with Bonferroni correction; Table 1).

Finally, the intergroup comparison showed no significant difference between the groups regarding Thongprasom baseline measurements (p>0.05 by the Mann-Whitney U test). On the other hand, the intragroup comparisons showed a statistically significant difference between all time measurements of the Thongprasom values in both the i-PRF group and the corticosteroid group (p<0.05 by the Friedman test). According to the *post-hoc* pairwise comparisons, the Thongprasom 1^st^, 2^nd^ and 6^th^ month median values were significantly lower in both groups compared to the baseline median values (p<0.008 by the Wilcoxon test with Bonferroni correction; Table 2).

**Table 2:** Comparisons of Thongprasom levels with intragroup and intergroup

		i-PRF Group	Corticosteroid Group	p1
		Mean±SD	Mean±SD	
		Med (Min-Max)	Med (Min-Max)	
Thongprasom	Baseline	4.79±0.41	4.71±0.46	0.509
		5 (4-5)	5 (4-5)	
	1^st^ month	1.88±1.3^a^	2±1.41^a^	0.720
		1 (0-5)	2 (0-5)	
	2^nd^ month	1.58±0.83^a^	1.88±1.23^a^	0.475
		1 (0-3)	1.5 (0-5)	
	6^th^ month	1.88±1.08^a^	2.21±1.35^a^	0.441
		1.5 (1-5)	2 (1-5)	
p*		<0.001	<0.001	

*Friedman test p-value (intragroup); statistical significance (p<0.05) marked in bold.

1Mann-Whitney U test p-value (intergroup).

a Statistical significance compare to baseline, using the Wilcoxon test with Bonferroni-correction (p<0.008).

Abbreviations: VAS, visual analog scale; i-PRF, injectable platelet-rich fibrin; SD, standard deviation; Med, median; Min, minimum; Max, maximum.

We observed no statistically significant difference between the groups regarding OHIP-14 measurements (p<0.05 by the Friedman test). According to the *post-hoc* pairwise comparisons, the median OHIP-14 values were significantly lower in all months compared to baseline values (p<0.008 by the Wilcoxon test with Bonferroni correction; Table 3). Moreover, the 2^nd^ month OHIP-14 measurements were significantly lower than the 1^st^ month measurements in the patients (p<0.008 by the Wilcoxon test with Bonferroni correction; Table 3).

**Table 3:** Comprasions of OHIP-14 values

		Mean±SD
		Med (Min-Max)
OHIP-14	Baseline	34.79±10.85
		33 (20-56)
	1^st^ month	20.04±7.29^a^
		19.5 (8-41)
	2^nd^ month	15.83±6.31^a,b^
		15 (4-26)
	6^th^ month	16.42±12.12^a^
		12 (4-48)
p*		<0.001

*Friedman test p-value (intragroup); statistical significance (p<0.05) marked in bold.

a Statistical significance compare to baseline,

b Statistical significance compare to 1st month, using the Wilcoxon test with Bonferroni-correction (p<0.008).

Abbreviations: VAS, visual analog scale; OHIP-14, Oral Health Impact Profile-14; SD, standard deviation; Med, median; Min, minimum; Max, maximum.

## Discussion

In our study, VAS-pain levels after the last injection in the i-PRF group were lower, and VAS-satisfaction levels were higher. However, the i-PRF and corticosteroid groups showed no difference regarding VAS-pain, VAS-satisfaction, or lesion size. In contrast, the 6^th^month VAS-pain and VAS-satisfaction values in the i-PRF group differed significantly from both the baseline levels and the levels after the last injection.

Topical corticosteroids can be used to minimize adverse drug effects, since they only affect the lesion and the surrounding tissues. This is the reason why topical corticosteroid administration is considered the first-choice treatment in OLP, rather than a systemic treatment.^26^ Intralesional applications may obtain a better response when topical corticosteroids are ineffective in resolving and healing the lesion.^9,20,26^ Studies have proven the effectiveness of intralesional corticosteroid applications in OLP management; however, their continuous and long-term use is associated with many systemic side effects.^27,28^ Thus, a need for a more effective and efficacious treatment of erosive OLP with few or no side effects emerges. Studies toward this goal has focused on the use of biomolecules, such as platelet-rich plasma (PRP) and platelet-rich growth factor (PRGF), which are rich in growth factors and act as a continuous release scaffold. The use of biomolecules to treat lichen planus is growing.^9,27,29,30^ In our study, we compared the effectiveness of intralesional application of Methylprednisolone acetate, which is a corticosteroid and i-PRF, an autologous factor, in bilateral EOLP lesions.

Oral health problems can cause pain and discomfort, and lead to problems with eating and drinking, social relations, appearance, and self-reliance. The OLP is a severe disease with symptoms and complications that affect individuals’ lives.^9,31,32^ Patients with have poor quality of life,^31^ and high VAS-pain values are associated with increased OHIP-14 score in individuals with EOLP.^33^ However, the VAS-pain and OHIP-14 values of individuals with OLP decrease after topical corticosteroid treatment.^32^ Ahuja, et al.^27^ (2020) compared intralesional PRP and triamcinolone acetonide applications in EOLP lesions by measuring changes in VAS-pain values during 4 months of follow-up. They reported that both applications were successful and had similar effectiveness. Likewise, Bennardo, et al.^30^ (2021) compared the 4-week results of PRF and triamcinolone acetonide injected therapies in patients with OLP patients in a split mouth study. The authors reported a mean decrease of 47.6% in the VAS score for PRF-treated sites; the decrease in the score for the triamcinolone acetonide–treated sites was 40%. The study reported no statistically significant difference between the groups; however, the authors stated that PRF is as effective as triamniconole acetonoide at reducing VAS-pain values for OLP lesions. Our study showed that baseline VAS-pain and OHIP-14 levels were high and that VAS-pain and OHIP-14 values decreased significantly at the 1^st^, 2^nd^, and 6^th^ months. Similarly, VAS-satisfaction values increased in both treatment groups compared to baseline values.

In previous studies, EOLP lesion sizes decreased significantly after topical or injected corticosteroid applications, as defined in terms of Thongprasom values.^16,30,32^ Studies have compared intralesional PRP and triamcinolone acetonide applications in EOLP lesions considering the changes in lesion sizes. PRP application has also provided efficacy similar to that of triamcinolone acetonide.^27^ In their split-mouth study of injectable treatments, Bennardo, et al.^30^ (2021) reported a mean decrease of 59.8% in the lesion size for PRF-treated sites and of 59.2% for triamniconole acetonoide-treated sites. Despite the inexistence of statistical difference between the groups regarding reduction in lesion size, the authors stated that PRF had effectiveness similar to that of triamcinolone acetonide. Corroborating these studies, our study showed a significant decrease in EOLP lesion size in both the i-PRF and corticosteroid groups at the 1^st^, 2^nd^ and 6^th^ months when compared with baseline.

Oral lichen planus lesions show higher expression levels of inflammatory cytokines, including toll-like receptor/nuclear factor-κB p65, IL-1β, IL-6 and tumor necrosis factor-α.^34^ Zhang, et al.^35^ (2020) reported that i-PRF can reduce the inflammatory response caused by lipopolysaccharides to some extent. The authors emphasized the potential anti-inflammatory role of i-PRF, since they found out that it inhibited the TLR4 (an inflammatory stimulation activator) and p-p65 (a key factor of the classical inflammatory-related NF-kB signaling pathway). Thus, the use of i-PRF may support healing by reducing the immune response and be an appropriate clinical strategy to alleviate the symptoms of OLP.^35^ Pinas, et al.^29^ (2018) found that OLP lesions may lack certain growth factors, which could be eliminated by local application of autologous factors (e.g., PRGF). The authors reported this application improved cell functions and restored cell-matrix communication. Like PRGF, i-PRF gradually releases growth factors, thus reducing inflammation in the environment, and these actions facilitate proper tissue healing and modification of the cellular environment.^8^ In our study, the positive effects of i-PRF on VAS-pain and VAS- satisfaction levels and on lesion sizes may be due to all these features.

We decided not to perform biopsy after the treatments, since a mechanical trauma can trigger new erosive lesions in lichen planus.^3^ This decision, however, impeeded the performance of histopathological evaluations. Therefore, we investigated the treatment results subjectively using the VAS and objectively using Thongprasom scoring. One limitation of our study is the fact that the systemic absorption of the corticosteroid on the contralateral lesion area has unknown side effects. However, our study used a split-mouth design, found in the literature,^30^ to eliminate patient-related factors and to compare lesions with similar characteristics. Another limitation of our study is the fluctuation of i-PRF doses resulting from imprecise nature of centrifugation.

## Conclusion

Within the limitations of our study, the pain and satisfaction values were positively affected by both i-PRF and corticosteroid treatments, and the quality of life increased after the procedures. These results suggest that i-PRF may be beneficial in the treatment of EOLP lesions. Although it cannot be considered a first-choice treatment, the i-PRF may be considered an alternative therapy in patients who are unresponsive to topical corticosteroids. Biochemical and histopathological studies should be conducted in larger populations to reveal the mechanism of i-PRF action in EOLP treatment.
